# Diuretic Intensity Score and Mortality in Hospitalized Heart Failure Patients: A Multicenter Propensity‐Matched Analysis

**DOI:** 10.1002/clc.70386

**Published:** 2026-06-19

**Authors:** Jianwei Zhou

**Affiliations:** ^1^ Department of Cardiology Guizhou Provincial People's Hospital Guiyang China

**Keywords:** diuretics, heart failure, intensive care units, mortality, propensity score matching, risk assessment

## Abstract

**Aims:**

To develop and evaluate a novel Diuretic Intensity Score (DIS) and investigate its relationship with mortality in hospitalized heart failure patients.

**Methods:**

We analyzed propensity‐matched data from two independent cohorts: MIMIC‐IV (*n* = 15 942) and the China Regional Heart Failure Database (*n* = 1884). DIS was calculated based on diuretic types, routes, and combinations. Primary and secondary outcomes were 28‐ and 90‐day all‐cause mortality.

**Results:**

Higher DIS was significantly associated with reduced 28‐day mortality (Severe vs. Mild: HR 0.27, *p* < 0.001 in MIMIC‐IV; HR 0.10, *p* < 0.001 in Chinese cohort) and 90‐day mortality. Restricted cubic spline analysis revealed an inverse, non‐linear relationship, with mortality risk decreasing as intensity increased and then plateauing.

**Conclusions:**

In this propensity‐matched analysis of two independent cohorts, a higher DIS was associated with lower 28‐ and 90‐day all‐cause mortality in hospitalized heart failure patients. Given the observational design, these findings should be regarded as hypothesis‐generating associations rather than evidence of a causal benefit and require confirmation in prospective randomized trials before any change in clinical practice can be recommended.

## Graphical abstract

In two independent cohorts of 17 826 hospitalized heart failure patients (MIMIC‐IV and a Chinese database), a higher Diuretic Intensity Score was associated with lower 28‐ and 90‐day all‐cause mortality. These findings are hypothesis‐generating and warrant validation in prospective randomized trials.

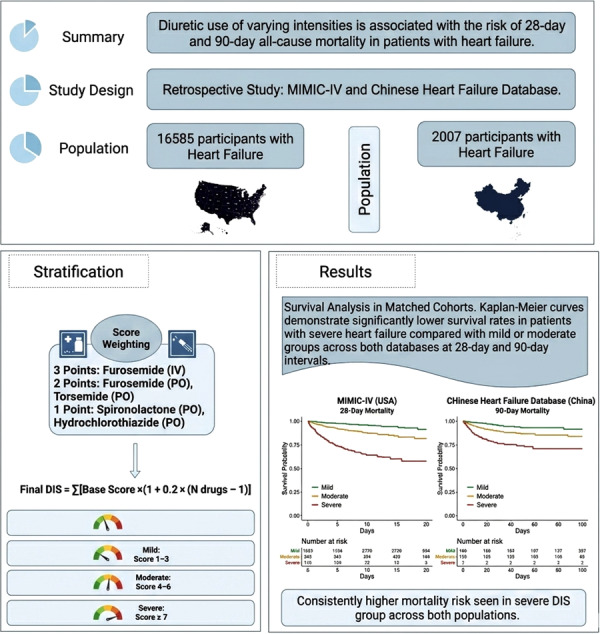

## 1 Introduction

Heart failure represents a major global public health challenge, affecting more than 64 million individuals worldwide [1], with continuously rising incidence and prevalence. Despite significant advances in heart failure therapeutics in recent years, short‐term and long‐term outcomes for hospitalized heart failure patients remain poor, with 28‐ and 90‐day mortality rates reaching 10%−15% and 20%−30%, respectively [2, 3]. Therefore, optimizing in‐hospital management strategies for heart failure is crucial for improving patient outcomes.

Loop diuretics constitute the cornerstone of acute heart failure management, with nearly all patients hospitalized for acute decompensated heart failure receiving diuretic therapy to relieve congestive symptoms [4]. However, considerable controversy persists regarding optimal diuretic utilization strategies, particularly concerning appropriate dose intensity. Current heart failure guidelines recommend using the lowest effective diuretic dose to achieve volume balance [5, 6], yet in clinical practice, diuretic use patterns vary widely, ranging from low‐dose intermittent administration to high‐dose continuous infusion [7].

Previous studies have yielded inconsistent results regarding the relationship between diuretic dosing and clinical outcomes. Some investigations suggest that high‐dose diuretics are associated with adverse outcomes, including worsening renal function, electrolyte disturbances, and increased mortality [8, 9]. This may result from neurohormonal activation, hemodynamic instability, and renal injury induced by high‐dose diuretics [7, 10]. Conversely, other studies argue that adequate diuresis and effective decongestion are essential for improving heart failure outcomes, and that insufficient diuresis may lead to persistent congestion, thereby increasing risks of rehospitalization and mortality [11, 12].

A key challenge currently is the lack of standardized methods to quantify and compare the intensity of different diuretic regimens. Different diuretics possess varying potencies, and multiple administration routes and dosing regimens are commonly employed clinically, making direct comparisons difficult [13]. Previous studies have predominantly utilized simple dose classifications (e.g., high‐dose vs. low‐dose) or focused solely on single diuretics, failing to comprehensively reflect the complexity of diuretic use in clinical practice. This methodological limitation may be an important factor contributing to the contradictory results in existing literature [14, 15].

Therefore, this study developed the Diuretic Intensity Score (DIS) system, aiming to provide a standardized tool for comprehensively evaluating the intensity of diuretic use during hospitalization. Through this innovative scoring system, we can more accurately quantify the intensity of different diuretic regimens and systematically assess their relationship with short‐term and intermediate‐term mortality following discharge in heart failure patients. By validating this hypothesis in two independent large‐scale real‐world databases (MIMIC‐IV and the Chinese Regional Heart Failure Database) and employing propensity score matching (PSM) methods to control for potential confounding factors, this study is expected to provide important evidence‐based medical data for optimizing diuretic utilization strategies in heart failure patients and offer a practical tool for assessing diuretic intensity in clinical practice.

## 2 Materials and Methods

### 2.1 Data Sources

In this study, we utilized two publicly accessible datasets: the MIMIC‐IV v3.1 dataset [16] and the China Regional Heart Failure Database generated at Zigong Fourth People's Hospital [17]. MIMIC‐IV contains deidentified data from 65 366 unique patients admitted to Beth Israel Deaconess Medical Center between 2008 and 2019 (single‐center dataset). The China Regional Heart Failure Database includes 2,008 patients with heart failure admitted to Zigong Fourth People's Hospital between December 2016 and June 2019. For the MIMIC database, the authors of this study maintained access privileges to the database granted by the relevant institution (certificate number 65254134). Our study was deemed exempt from ethical review by the institutional review board.

Study population

The MIMIC‐IV critical care database was screened using strict inclusion and exclusion criteria, ultimately enrolling eligible heart failure patients. Exclusion criteria included: (1) patients younger than 18 years; (2) patients with non‐initial ICU admissions. The China Regional Heart Failure Database applied the same inclusion criteria as MIMIC‐IV to ensure comparability between groups. Exclusion criteria were: (1) patients younger than 18 years; (2) patients with missing medication data.

### 2.2 Construction of the DIS

To quantify the intensity of diuretic therapy, we developed the DIS system based on the following principles:

Five diuretics were assigned scores based on their pharmacological properties and clinical efficacy: furosemide injection (intravenous) 3 points, furosemide tablet 2 points, torsemide tablet 2 points, spironolactone tablet 1 point, and hydrochlorothiazide tablet 1 point. The scoring rationale includes: (1) loop diuretics (furosemide, torsemide) have greater diuretic potency than thiazide or potassium‐sparing diuretics; (2) intravenous administration, typically used in acute decompensation or severe volume overload, warrants a higher score; (3) potassium‐sparing and thiazide diuretics, often used as adjunctive therapy, receive lower scores.

The DIS employs a weighted algorithm that accounts for both individual drug efficacy and the impact of combination therapy: Base score = Σ(drug use status × corresponding score); Number of drugs used = Σ(drug use status); Weighted DIS = Base score × (1 + 0.2 × [number of drugs used − 1]). Here, drug use status is a binary variable (1 = used, 0 = not used). The weighting factor of 0.2 reflects a 20% increase in treatment intensity per additional diuretic, based on clinical observations that combination therapy often indicates diuretic resistance or more severe volume overload. This 20% increment was a pragmatic, clinically informed choice rather than an empirically estimated coefficient; because no external gold standard exists for this weight, we examined the sensitivity of our findings to it by re‐deriving the DIS using alternative increments (0%, i.e., an unweighted sum; 10%; and 30%) and by comparing the weighted DIS with a simple unweighted count of diuretic agents.

Based on the DIS, diuretic therapy intensity is classified into three levels: mild (1−3 points), typically single‐drug or mild combination therapy; moderate (4−6 points), loop diuretics combined with other diuretics; and severe (≥ 7 points), intravenous loop diuretics or multiple‐drug combinations. This scoring system provides a standardized metric for quantifying diuretic treatment intensity in subsequent prognostic analyses.

### 2.3 Outcome Variable

The primary outcome of this study was 28‐day mortality, with 90‐day mortality as the secondary outcome. All outcome data were complete, with no missing values. Mortality status was defined using binary coding: 0 for survival and 1 for death. Additionally, detailed survival time data (days from admission to death or study endpoint) were collected, with no missing values.

### 2.4 Covariates

The China Regional Heart Failure Database collected covariates including demographic characteristics (age and sex), physiological parameters (systolic blood pressure, diastolic blood pressure, and body mass index [BMI]), key laboratory measures (serum creatinine, serum potassium, B‐type natriuretic peptide [BNP]), and major comorbidities (history of myocardial infarction, cerebrovascular disease, chronic obstructive pulmonary disease, diabetes, and chronic kidney disease). The MIMIC‐IV database included the same covariates (based on initial ICU admission measurements). However, due to high missing rates (> 50%) for BMI and BNP in MIMIC‐IV, these variables were excluded from statistical analyses to ensure the reliability of results and comparability between the two databases.

### 2.5 Statistical Analysis

In this study, missing data accounted for less than 5% of the total. To minimize the risk of selection bias due to missing data, multiple imputation by chained equations was employed [18]. Continuous variables were presented as median (interquartile range, IQR) and analyzed using the Kruskal−Wallis test. Categorical variables were expressed as frequencies (percentages) and compared using the chi‐square test or Fisher's exact test. To estimate the hazard ratios (HR) and corresponding 95% confidence intervals (CI) for the association between DIS and 28‐day and 90‐day mortality, multivariable Cox proportional hazards regression models were developed. In the China Regional Heart Failure database, Model 1 was unadjusted. Model 2 was adjusted for sex, BMI, age, diastolic blood pressure, systolic blood pressure, serum potassium, BNP, and creatinine. Model 3 was further adjusted for comorbidities including myocardial infarction, cerebrovascular disease, chronic obstructive pulmonary disease, diabetes mellitus, and chronic kidney disease, Given the limited number of events in the Chinese cohort (*n* = 35 at 28 days), Model 3 in this cohort should be interpreted as exploratory, as the events‐per‐variable ratio falls below the conventional threshold of 10; the corresponding effect estimates are therefore reported with appropriate caveats in the Results and Discussion. For the MIMIC‐IV database, Model 1 was unadjusted. Model 2 was adjusted for sex, age, diastolic blood pressure, systolic blood pressure, serum potassium, and creatinine. Model 3 was further adjusted for comorbidities including myocardial infarction, cerebrovascular disease, chronic obstructive pulmonary disease, diabetes mellitus, and chronic kidney disease. Cumulative incidence rates of 28‐ and 90‐day mortality were calculated and compared using the Kaplan−Meier method and log‐rank test. All analyses were two‐sided with *p* < 0.05 considered statistically significant. Statistical analyses were performed using R software (version 4.2.3; R Foundation for Statistical Computing, Vienna, Austria).

To evaluate the incremental prognostic value of the DIS beyond simpler diuretic metrics, we additionally compared three nested Cox proportional hazards models: (i) baseline covariates only; (ii) covariates plus a simple comparator metric (intravenous furosemide use as a binary indicator, or an unweighted count of diuretic types); and (iii) covariates plus the DIS. Model discrimination was assessed by Harrell's C‐index, and incremental discrimination was quantified by the continuous Net Reclassification Improvement (NRI), Integrated Discrimination Improvement (IDI), and likelihood ratio tests. To assess robustness to unmeasured confounding, we additionally calculated E‐values for the observed DIS–mortality associations. The E‐value represents the minimum strength of association (on the risk‐ratio scale) that an unmeasured confounder would need to have with both the exposure and the outcome to fully explain the observed effect.

### 2.6 PSM

This study utilized PSM to process three data groups, aiming to minimize intergroup covariate imbalances [19]. Matching was based on diuretic use intensity levels, with covariates including age, sex, systolic, and diastolic blood pressure, BMI, serum creatinine, serum potassium, BNP, and medical history (myocardial infarction, cerebrovascular disease, chronic obstructive pulmonary disease, diabetes, and chronic kidney disease). In the MIMIC‐IV database, all covariates except BMI and BNP were consistent with those in the China Regional Heart Failure Database. Matching effectiveness was assessed using the standardized mean difference (SMD), with SMD < 0.1 indicating good balance [20].

### 2.7 Subgroup Analyses

To evaluate the impact of different variables on 28‐ and 90‐day all‐cause mortality in heart failure patients, subgroup analyses were conducted in both databases based on the following variables: sex, history of myocardial infarction, chronic obstructive pulmonary disease, moderate‐to‐severe chronic kidney disease, and age (< 70 vs. ≥ 70  years).

## 3 Result

### 3.1 Patient Selection and PSM

**Figure 1:**
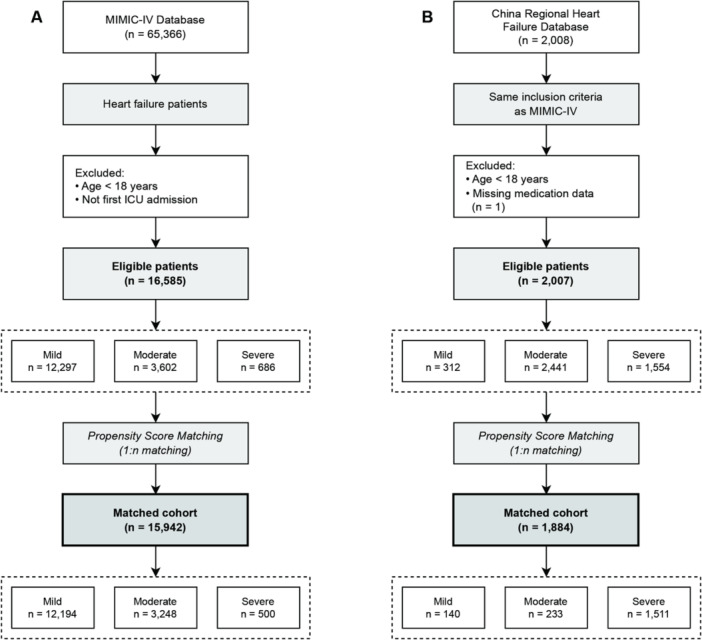
Patient selection and propensity score matching flowcharts for (A) Medical Information Mart for Intensive Care IV (MIMIC‐IV) Database and (B) China Regional Heart Failure Database. Flow diagrams demonstrate the patient selection process from initial populations of 65 366 and 2008 patients, respectively. Exclusion criteria included age less than 18 years, not first intensive care unit admission for MIMIC‐IV, and missing medication data for the China Regional Heart Failure Database. Eligible patients were stratified by heart failure severity (mild, moderate, severe) and underwent 1:*n* propensity score matching, resulting in final matched cohorts of 15 942 patients from MIMIC‐IV and 1884 patients from the China Regional Heart Failure Database.

**Figure 2:**
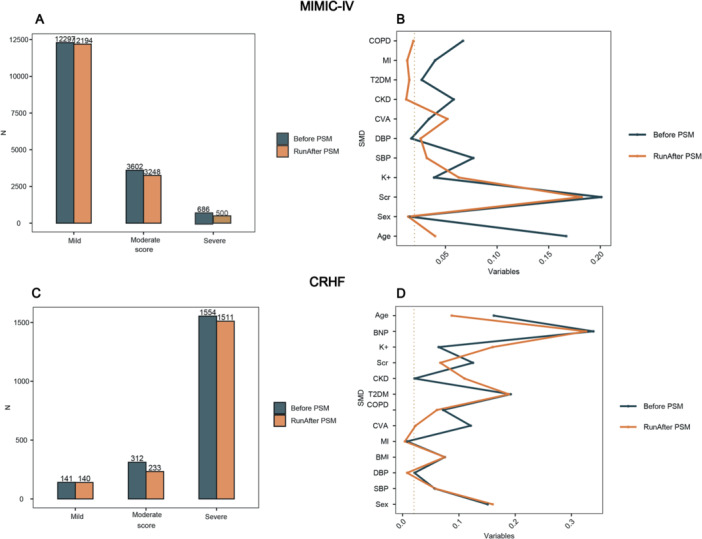
Baseline characteristics and propensity score matching assessment comparing Medical Information Mart for Intensive Care IV (MIMIC‐IV) and China Regional Heart Failure databases. (A) Distribution of patients by heart failure severity in the MIMIC‐IV database before and after propensity score matching. (B) Standardized mean differences for clinical variables before and after propensity score matching in the MIMIC‐IV database, with dashed vertical lines indicating standardized difference thresholds of 0.1. (C) Distribution of patients by heart failure severity in the China Regional Heart Failure database before and after propensity score matching. (D) Standardized mean differences for clinical variables before and after propensity score matching in the China Regional Heart Failure database. Variables include chronic obstructive pulmonary disease, myocardial infarction, type 2 diabetes mellitus, chronic kidney disease, cerebrovascular accident, diastolic and systolic blood pressure, serum potassium, serum creatinine, B‐type natriuretic peptide, body mass index, age, and sex.

From the MIMIC‐IV database, 16 585 eligible heart failure patients were identified after excluding patients younger than 18 years and those with non‐first ICU admissions from the initial cohort of 65 366 patients. The China Regional Heart Failure Database contributed 2007 eligible patients after applying identical inclusion criteria and excluding 1 patient with missing medication data. Following PSM, the MIMIC‐IV cohort was reduced to 15 942 patients (mild: *n* = 12 194; moderate: *n* = 3248; severe: *n* = 500), while the Chinese cohort included 1884 patients (mild: *n* = 140; moderate: *n* = 233; severe: *n* = 1511), as illustrated in Figure 1 and Figures 2A,C. The final analysis included 15 942 patients from MIMIC‐IV and 1884 patients from the Chinese database. Love plots demonstrated successful covariate balance, with SMDs less than 0.1 for most variables after matching (Figures 2B,D), indicating effective reduction of baseline differences between these two geographically distinct populations.

### 3.2 Patient Baseline Characteristics in MIMIC‐IV and Chinese Heart Failure Cohorts

Before PSM in the MIMIC‐IV database, patients with different heart failure severities exhibited significant differences in age distribution, serum creatinine levels, and chronic kidney disease prevalence (all *p* < 0.001). After matching, baseline characteristics achieved effective balance across groups, with SMDs reduced to below 0.1. Baseline characteristics of the MIMIC‐IV cohort are detailed in Table 1.

**Table 1:** Baseline characteristics in MIMIC‐IV before and after propensity score matching.

	Before propensity score matching total (*N* = 16 585)					After propensity score matching (*N* = 15 942)				
Variable	Mild (*N* = 12 297)	Moderate (*N* = 3602)	Severe (*N* = 686)	*p* value	SMD	Mild (*N* = 12 194)	Moderate (*N* = 3248)	Severe (*N* = 500)	*p* value	SMD
Age, years, n (%)				< 0.001	0.167				0.388	0.040
< 70	8169.00 (66.43%)	2341.00 (64.99%)	372.00 (54.23%)			8132.00 (66.69%)	2153.00 (66.29%)	319.00 (63.80%)		
≥ 70	4128.00 (33.57%)	1261.00 (35.01%)	314.00 (45.77%)			4062.00 (33.31%)	1095.00 (33.71%)	181.00 (36.20%)		
Sex, n (%)				0.872	0.015				0.847	0.014
Female	5472.00 (44.50%)	1609.00 (44.67%)	299.00 (43.59%)			5433.00 (44.55%)	1463.00 (45.04%)	220.00 (44.00%)		
Male	6825.00 (55.50%)	1993.00 (55.33%)	387.00 (56.41%)			6761.00 (55.45%)	1785.00 (54.96%)	280.00 (56.00%)		
SBP (mmHg)	119.00 (103.00−137.00)	118.00 (103.00−135.00)	116.00 (101.00−132.00)	0.008	0.077	119.00 (103.00−137.00)	118.00 (103.00−134.00)	117.00 (104.00−133.00)	0.110	0.032
DBP (mmHg)	65.00 (55.00−78.00)	64.00 (54.00−78.00)	66.00 (55.00−79.00)	0.325	0.017	65.00 (55.00−78.00)	64.00 (55.00−77.00)	65.50 (56.00−77.50)	0.288	0.026
Serum creatinine (μmol/L)	1.20 (0.90−2.10)	1.20 (0.90−1.70)	1.20 (0.90−1.70)	< 0.001	0.201	1.20 (0.90−2.00)	1.20 (0.90−1.70)	1.20 (0.90−1.80)	< 0.001	0.182
Potassium (mmol/L)	4.20 (3.80−4.70)	4.20 (3.80−4.70)	4.20 (3.80−4.70)	0.401	0.039	4.20 (3.80−4.70)	4.20 (3.80−4.60)	4.20 (3.80−4.60)	0.007	0.063
MI history, *n* (%)	1928.00 (15.68%)	562.00 (15.60%)	93.00 (13.56%)	0.328	0.04	1910.00 (15.66%)	490.00 (15.09%)	79.00 (15.80%)	0.713	0.013
Cerebrovascular disease, *n* (%)	1450.00 (11.79%)	377.00 (10.47%)	70.00 (10.20%)	0.052	0.034	1438.00 (11.79%)	338.00 (10.41%)	47.00 (9.40%)	0.031	0.052
COPD, *n* (%)	2804.00 (22.80%)	913.00 (25.35%)	145.00 (21.14%)	0.003	0.067	2794.00 (22.91%)	760.00 (23.40%)	111.00 (22.20%)	0.769	0.019
Diabetes, *n* (%)	4819.00 (39.19%)	1482.00 (41.14%)	271.00 (39.50%)	0.108	0.027	4792.00 (39.30%)	1270.00 (39.10%)	201.00 (40.20%)	0.895	0.015
CKD, *n* (%)	4216.00 (34.28%)	1384.00 (38.42%)	235.00 (34.26%)	< 0.001	0.058	4213.00 (34.55%)	1151.00 (35.44%)	175.00 (35.00%)	0.636	0.012

*Note:* Severity groups were defined as: Mild (1−3 points), Moderate (3−6 points), and Severe (≥ 7 points). Continuous variables are presented as median (IQR: Q1−Q3). Categorical variables are presented as *n* (%). *p* values were calculated using the Kruskal−Wallis test for continuous variables and the chi‐square test or Fisher's exact test for categorical variables. SMD was used to assess balance between groups, with SMD < 0.1 indicating good balance.

Abbreviations: CKD, chronic kidney disease; COPD, chronic obstructive pulmonary disease; DBP, diastolic blood pressure; IQR, interquartile range; MI, myocardial infarction; SBP, systolic blood pressure; SMD, standardized mean difference.

Before PSM in the China Regional Heart Failure Database, patients with different heart failure severities demonstrated significant differences in age distribution, sex composition, BNP levels, and diabetes prevalence (all *p* < 0.05). After matching, between‐group differences were effectively controlled for all variables except BNP. Baseline characteristics of the Chinese cohort are presented in Table 2.

**Table 2:** Baseline characteristics in the China Regional Heart Failure Database before and after propensity score matching.

	Before propensity score matching Total (*N* = 2007)					After propensity score matching (*N* = 1884)				
Variable	Mild (*N* = 312)	Moderate (*N* = 2441)	Severe (*N* = 1554)	*p* value	SMD	Mild (*N* = 140)	Moderate (*N* = 233)	Severe (*N* = 1511)	*p* value	SMD
Age, years, n (%)				< 0.001	0.162				0.192	0.087
< 70	101.00 (71.63)	254.00 (81.41)	1106.00 (71.17)			101.00 (72.14%)	181.00 (77.68%)	1088.00 (72.01%)		
≥ 70	40.00 (28.37)	58.00 (18.59)	448.00 (28.83)			39.00 (27.86%)	52.00 (22.32%)	423.00 (27.99%)		
Sex, n (%)				0.019	0.151				0.023	0.160
Female	95.00 (67.38%)	191.00 (61.22%)	877.00 (56.44%)			95.00 (67.86%)	139.00 (59.66%)	850.00 (56.25%)		
Male	46.00 (32.62%)	121.00 (38.78%)	677.00 (43.56%)			45.00 (32.14%)	94.00 (40.34%)	661.00 (43.75%)		
SBP (mmHg)	130.00 (120.00−148.00)	130.00 (120.00−149.00)	130.00 (111.00−146.00)	0.114	0.057	130.00 (120.00−149.00)	130.00 (118.00−148.00)	130.00 (112.00−146.00)	0.261	0.058
DBP (mmHg)	78.00 (69.00−86.00)	78.00 (67.00−86.00)	76.00 (65.00−84.00)	0.521	0.021	78.00 (68.50−86.00)	76.00 (64.00−86.00)	76.00 (65.00−84.00)	0.650	0.021
BMI (kg/m^2^)	20.55 (18.29−24.03)	20.77 (18.37−23.50)	20.76 (18.52−23.44)	0.917	0.075	20.55 (18.25−24.13)	20.89 (18.73−23.46)	20.76 (18.49−23.44)	0.980	0.075
Serum creatinine (μmol/L)	80.40 (61.90−107.10)	87.05 (64.80−128.65)	87.70 (65.20−122.60)	0.078	0.125	80.65 (61.70−108.45)	85.50 (64.60−123.00)	88.10 (65.40−123.80)	0.115	0.057
Potassium (mmol/L)	3.89 (3.56−4.38)	3.87 (3.55−4.34)	3.88 (3.51−4.32)	0.680	0.064	3.89 (3.58−4.41)	3.78 (3.49−4.23)	3.88 (3.52−4.32)	0.072	0.160
BNP (pg/mL)	250.82 (114.00−680.78)	481.63 (189.50−1106.58)	907.96 (388.69−1924.73)	< 0.001	0.339	250.88 (115.85−693.49)	549.95 (271.60−1127.16)	886.46 (378.67−1887.70)	< 0.001	0.326
MI history, *n* (%)	10.00 (7.09%)	23.00 (7.37%)	110.00 (7.08%)	0.983	0.008	10.00 (7.14%)	17.00 (7.30%)	110.00 (7.28%)	0.998	0.004
Cerebrovascular disease, *n* (%)	9.00 (6.38%)	36.00 (11.54%)	105.00 (6.76%)	0.012	0.121	9.00 (6.43%)	17.00 (7.30%)	105.00 (6.95%)	0.950	0.023
COPD, *n* (%)	13.00 (9.22%)	28.00 (8.97%)	191.00 (12.29%)	0.165	0.072	13.00 (9.29%)	22.00 (9.44%)	183.00 (12.11%)	0.337	0.063
Diabetes, *n* (%)	34.00 (24.11%)	43.00 (13.78%)	389.00 (25.03%)	< 0.001	0.192	34.00 (24.29%)	31.00 (13.30%)	352.00 (23.30%)	0.002	0.189
CKD, *n* (%)	34.00 (24.11%)	71.00 (22.76%)	369.00 (23.75%)	0.922	0.021	34.00 (24.29%)	41.00 (17.60%)	362.00 (23.96%)	0.096	0.110

*Note:* Severity groups were defined as: Mild (1−3 points), Moderate (3−6 points), and Severe (≥ 7 points). Continuous variables are presented as median (IQR: Q1−Q3). Categorical variables are presented as *n* (%). *p* values were calculated using the Kruskal−Wallis test for continuous variables and the chi‐square test or Fisher's exact test for categorical variables. SMD was used to assess balance between groups, with SMD < 0.1 indicating good balance.

Abbreviations: BMI, body mass index; BNP, B‐type natriuretic peptide; CKD, chronic kidney disease; COPD, Chronic Obstructive Pulmonary Disease; DBP, diastolic blood pressure; IQR, interquartile range; MI, Myocardial Infarction; SBP, systolic blood pressure; SMD, standardized mean difference.

### 3.3 Survival Analysis of DIS and Mortality

The results of univariable and multivariable Cox proportional hazards models for 28‐ and 90‐day all‐cause mortality are shown in Tables 3 and 4. As shown in Table 3, a higher DIS was associated with a lower observed risk of 28‐day all‐cause mortality in both cohorts.

**Table 3:** Association between DIuretic Intensity Score (DIS) and 28‐day all‐cause mortality in Cox proportional hazards models.

DIS	HR (95% CI), *p* value		
	Model 1	Model 2	Model 3
CRHF‐DB			
Continuous	0.82 (0.74–0.91), < 0.001	0.79 (0.71–0.89), < 0.001	0.79 (0.70–0.89), < 0.001
*Categories*			
Mild	Reference	Reference	Reference
Moderate	0.36 (0.15–0.86), 0.022	0.46 (0.18–1.19), 0.110	0.44 (0.17–1.16), 0.098
Severe	0.10 (0.05–0.20), < 0.001	0.10 (0.04–0.23), < 0.001	0.10 (0.04–0.22), < 0.001
MIMIC‐IV			
Continuous	0.86 (0.84–0.87), < 0.001	0.86 (0.84–0.88), < 0.001	0.86 (0.84–0.87), < 0.001
*Categories*			
Mild	Reference	Reference	Reference
Moderate	0.31 (0.27–0.36), < 0.001	0.32 (0.27–0.37), < 0.001	0.31 (0.27–0.37), < 0.001
Severe	0.26 (0.17–0.39), < 0.001	0.27 (0.18–0.41), < 0.001	0.27 (0.17–0.40), < 0.001

*Note:* Given the limited number of events in the CRHF‐DB cohort (*n* = 35 at 28 days), Model 3 estimates from the Chinese cohort should be interpreted as exploratory. CRHF‐DB cohort: Model 1: Unadjusted. Model 2: Adjusted for sex, BMI, age, diastolic blood pressure, systolic blood pressure, serum potassium, BNP, and serum creatinine. Model 3: Additionally adjusted for myocardial infarction, cerebrovascular disease, chronic obstructive pulmonary disease, diabetes mellitus, and chronic kidney disease. MIMIC‐IV cohort: Model 1: Unadjusted. Model 2: Adjusted for sex, age, diastolic blood pressure, systolic blood pressure, serum potassium, and serum creatinine. Model 3: Additionally adjusted for myocardial infarction, cerebrovascular disease, chronic obstructive pulmonary disease, diabetes mellitus, and chronic kidney disease.

Abbreviations: BMI, body mass index; BNP, B‐type natriuretic peptide; CI, confidence interval; CRHF‐DB, China Regional Heart Failure Database; HR, hazard ratio; MIMIC‐IV, Medical Information Mart for Intensive Care IV.

**Table 4:** Association between Diuretic Intensity Score (DIS) and 90‐day all‐cause mortality in Cox proportional hazards models.

DIS	HR (95% CI), *p* value		
	Model 1	Model 2	Model 3
CRHF‐DB			
Continuous	0.86 (0.77–0.95), 0.003	0.83 (0.74–0.93), 0.001	0.83 (0.74–0.93), 0.001
*Categories*			
Mild	Reference	Reference	Reference
Moderate	0.36 (0.15–0.86), 0.022	0.45 (0.17–1.14), 0.091	0.44 (0.17–1.12), 0.086
Severe	0.12 (0.06–0.25), < 0.001	0.12 (0.06–0.26), < 0.001	0.12 (0.05–0.26), < 0.001
MIMIC‐IV			
Continuous	0.87 (0.86–0.89), < 0.001	0.88 (0.86–0.89), < 0.001	0.87 (0.86–0.89), < 0.001
*Categories*			
Mild	Reference	Reference	Reference
Moderate	0.38 (0.34–0.44), < 0.001	0.39 (0.34–0.45), < 0.001	0.39 (0.34–0.44), < 0.001
Severe	0.32 (0.23–0.45), < 0.001	0.33 (0.24–0.47), < 0.001	0.33 (0.23–0.47), < 0.001

*Note:* Given the limited number of events in the CRHF‐DB cohort (*n* = 39 at 90 days), Model 3 estimates from the Chinese cohort should be interpreted as exploratory. CRHF‐DB cohort: Model 1: Unadjusted. Model 2: Adjusted for sex, BMI, age, diastolic blood pressure, systolic blood pressure, serum potassium, BNP, and serum creatinine. Model 3: Additionally adjusted for myocardial infarction, cerebrovascular disease, chronic obstructive pulmonary disease, diabetes mellitus, and chronic kidney disease. MIMIC‐IV cohort: Model 1: Unadjusted. Model 2: Adjusted for sex, age, diastolic blood pressure, systolic blood pressure, serum potassium, and serum creatinine. Model 3: Additionally adjusted for myocardial infarction, cerebrovascular disease, chronic obstructive pulmonary disease, diabetes mellitus, and chronic kidney disease.

Abbreviations: BMI, body mass index; BNP, B‐type natriuretic peptide; CI, confidence interval; CRHF‐DB, China Regional Heart Failure Database; HR, hazard ratio; MIMIC‐IV, Medical Information Mart for Intensive Care IV.

In the CRHF‐DB cohort, compared with the mild group, the severe group showed the lowest mortality risk in the fully adjusted model (Model 3) (HR, 0.10; 95% CI, 0.04–0.22; *p* < 0.001). However, given the limited number of events (*n* = 35), this Model 3 estimate from the Chinese cohort should be interpreted as exploratory. Similarly, in the MIMIC‐IV cohort, the severe group had significantly reduced mortality risk (HR, 0.27; 95% CI, 0.174−0.404; *p* < 0.001).

As shown in Table 4, 90‐day all‐cause mortality demonstrated similar trends. In the multivariable Cox proportional hazards model for the CRHF‐DB cohort, the severe group continued to show the lowest HR (HR, 0.12; 95% CI, 0.05−0.26; *p* < 0.001). The MIMIC‐IV cohort yielded consistent results, with the severe group showing significantly reduced 90‐day mortality risk (HR, 0.33; 95% CI, 0.23−0.47; *p* < 0.001). Notably, when DIS was analyzed as a continuous variable, each unit increase in DIS was associated with reduced mortality risk in both cohorts (CRHF‐DB: HR, 0.83; 95% CI, 0.74−0.93; *p* = 0.001; MIMIC‐IV: HR, 0.87; 95% CI, 0.86‐0.89; *p* < 0.001).

**Figure 3:**
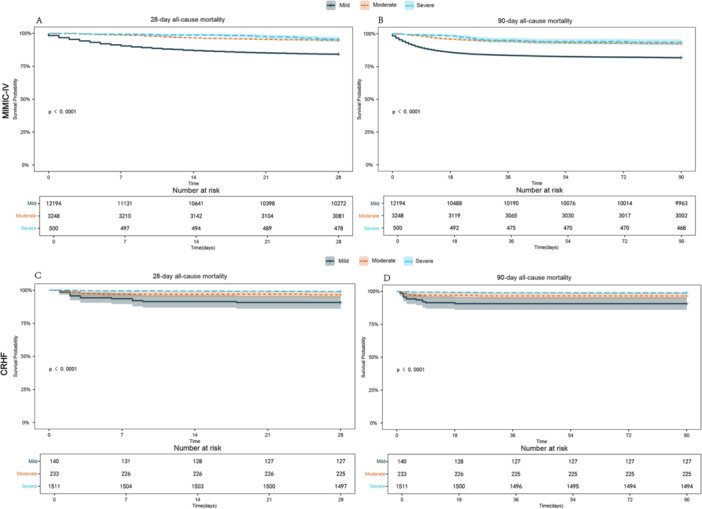
Kaplan−Meier survival curves for all‐cause mortality stratified by heart failure severity in matched cohorts. (A) 28‐day all‐cause mortality in Medical Information Mart for Intensive Care IV database (*n* = 15 942). (B) 90‐day all‐cause mortality in Medical Information Mart for Intensive Care IV database. (C) 28‐day all‐cause mortality in the China Regional Heart Failure database (*n* = 1884). (D) 90‐day all‐cause mortality in the China Regional Heart Failure database. Survival curves are displayed for mild (gray), moderate (orange), and severe (light blue) heart failure groups. *p* values less than 0.0001 indicate statistically significant differences between severity groups. Numbers at risk are shown at specified time intervals below each panel, with 95% confidence intervals represented by shaded areas.

Kaplan−Meier survival curves for 28‐ and 90‐day all‐cause mortality across different DIS categories (mild, moderate, and severe) are shown in Figure 3. In the MIMIC‐IV cohort, there were significant differences in both 28‐day (Figure 3A) and 90‐day (Figure 3B) all‐cause mortality among the three groups (both *p* < 0.0001). Similarly, in the CRHF cohort, significant differences were observed in 28‐day (Figure 3C) and 90‐day (Figure 3D) all‐cause mortality among the three groups (both *p* < 0.0001).

### 3.4 Dose‐Response Relationship of DIS

**Figure 4:**
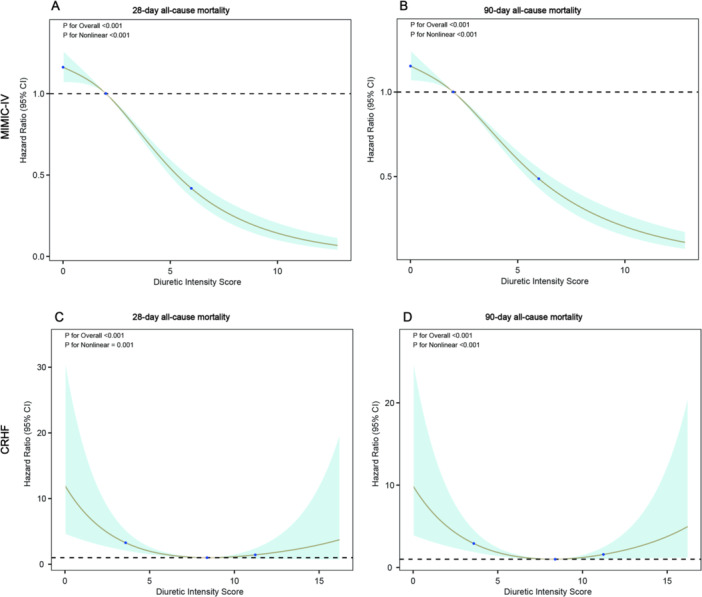
Restricted cubic spline analysis of the dose‐response relationship between Diuretic Intensity Score (DIS) and all‐cause mortality. (A) 28‐day all‐cause mortality in the MIMIC‐IV database (*n* = 15 942). (B) 90‐day all‐cause mortality in the MIMIC‐IV database. (C) 28‐day all‐cause mortality in the China Regional Heart Failure database (*n* = 1884). (D) 90‐day all‐cause mortality in the China Regional Heart Failure database. Curves show the hazard ratio (solid line) with 95% confidence interval (shaded area) as a function of continuous DIS. Models were adjusted for age, sex, blood pressure, serum creatinine, serum potassium, and major comorbidities. *p* values for the overall and non‐linear associations are reported within each panel.

Restricted cubic spline analysis revealed a nonlinear association between DIS and all‐cause mortality in both cohorts. In the MIMIC‐IV cohort, the risk of 28‐day (*p* for overall < 0.001; *p* for nonlinear < 0.001) (Figure 4A) and 90‐day (*p* for overall < 0.001; *p* for nonlinear < 0.001) (Figure 4B) all‐cause mortality decreased in a non‐linear, dose‐dependent pattern with increasing DIS. Similar nonlinear associations were observed in the CRHF cohort, with both 28‐day (*p* for overall < 0.001; *p* for nonlinear = 0.001) (Figure 4C) and 90‐day (*p* for overall < 0.001; *p* for nonlinear < 0.001) (Figure 4D) all‐cause mortality risk decreasing with increasing DIS, reaching a nadir at scores between approximately 5 and 10, and then plateauing.

### 3.5 Incremental Value of DIS

To assess whether the DIS provides incremental prognostic information beyond simpler diuretic metrics, we compared nested Cox models in both cohorts. In the MIMIC‐IV cohort, the categorical DIS (mild/moderate/severe) yielded C‐statistics of 0.677 and 0.665 for 28‐ and 90‐day mortality, respectively, which were comparable to or marginally higher than those of the continuous DIS (0.667 and 0.656), supporting the use of the three‐tier classification in the primary analysis. In the Chinese cohort, the DIS provided modest discrimination improvement over intravenous furosemide use alone (ΔC‐index +0.049 for 28‐day and +0.023 for 90‐day mortality; continuous NRI +0.836 and +0.707; IDI +0.054 and +0.027). When compared with the simple unweighted count of diuretic types, however, the DIS did not show superior discrimination (ΔC‐index −0.016 and −0.012), suggesting that part of the prognostic information captured by the weighted DIS overlaps with the number of agents prescribed. These exploratory comparisons should be interpreted with caution given the small number of events in the Chinese cohort (*n* = 35–39). The direction and statistical significance of the DIS–mortality associations were also preserved under alternative weighting increments (0%, 10%, and 30%), and the comparable performance of the simple unweighted count indicates that our conclusions do not hinge on the specific choice of weighting factor.

### 3.6 Robustness to Unmeasured Confounding

To quantify how strong an unmeasured confounder would need to be to fully explain the observed DIS–mortality association, we calculated E‐values from the per‐unit hazard ratios of the continuous DIS. In the MIMIC‐IV cohort, point‐estimate E‐values were 1.61 for 28‐day mortality and 1.55 for 90‐day mortality, with lower‐bound E‐values of 1.55 and 1.50, respectively, indicating moderate robustness to unmeasured confounding. In the Chinese cohort, point‐estimate E‐values were lower (1.21–1.24), with lower bounds approaching 1.00, reflecting greater susceptibility to residual confounding in this smaller dataset.

### 3.7 Subgroup Analysis

**Figure 5:**
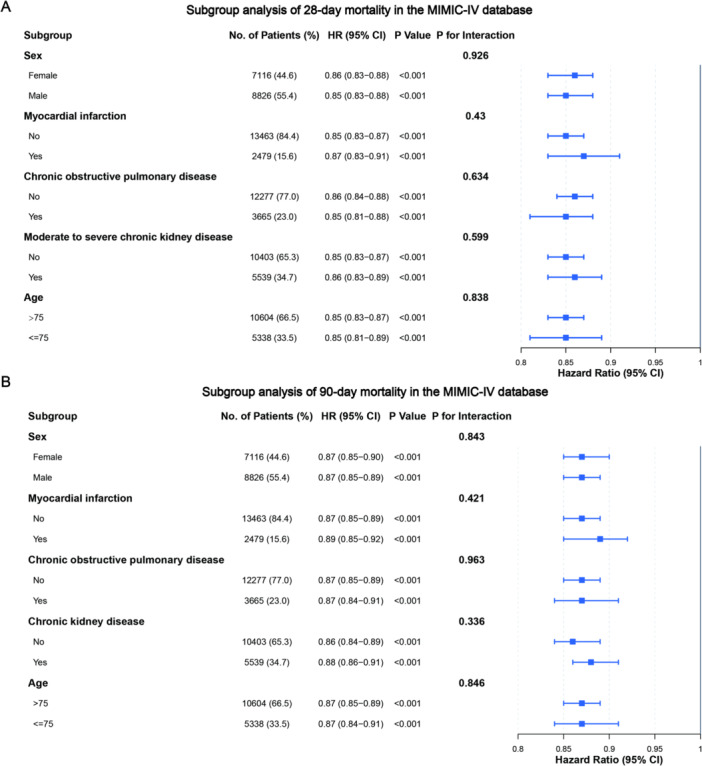
Subgroup analysis of the association between Diuretic Intensity Score and short‐term mortality in the MIMIC‐IV database. (A) 28‐day mortality; (B) 90‐day mortality. Adjusted for age, sex, myocardial infarction, chronic obstructive pulmonary disease, chronic kidney disease, and other baseline characteristics. Subgroup analyses were stratified by sex, myocardial infarction, chronic obstructive pulmonary disease, chronic kidney disease, and age (< 70 or ≥ 70 years). Hazard ratios (HRs) and 95% confidence intervals (CIs) were estimated using multivariable Cox proportional hazards models. No significant interactions were observed across subgroups (all *p* for interaction > 0.3).

**Figure 6:**
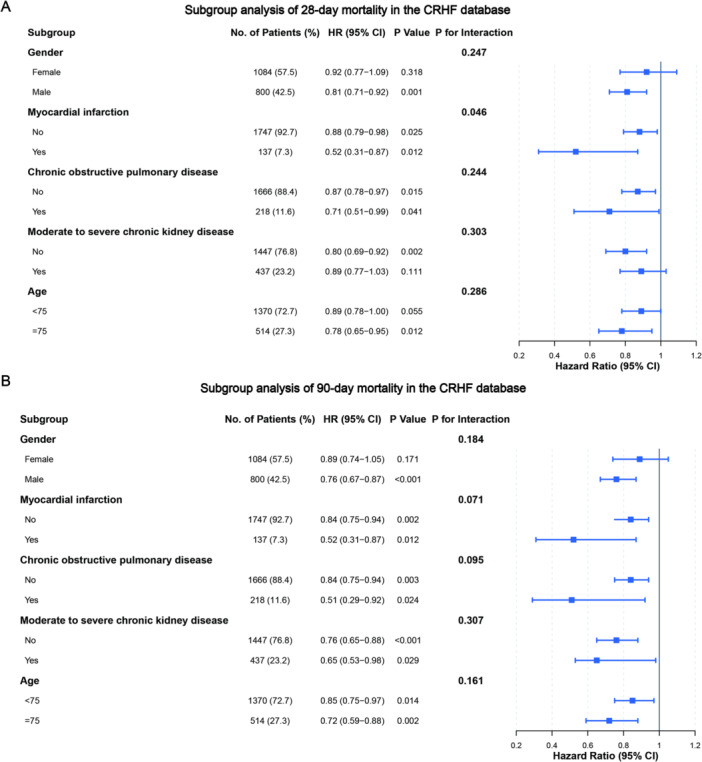
Subgroup analysis of the association between Diuretic Intensity Score and short‐term mortality in the CRHF database. (A) 28‐day mortality; (B) 90‐day mortality. Adjusted for age, gender, myocardial infarction, chronic obstructive pulmonary disease, moderate to severe chronic kidney disease, and other relevant clinical characteristics. Subgroup analyses were stratified by gender, myocardial infarction, chronic obstructive pulmonary disease, chronic kidney disease, and age (< 70 or ≥ 70 years). Hazard ratios (HRs) and 95% confidence intervals (CIs) were estimated using multivariable Cox proportional hazards models. Interaction P values were calculated to assess effect modification. A significant interaction was observed for myocardial infarction in the 28‐day mortality model (*p* for interaction = 0.046), while other subgroup interactions were not statistically significant (all *p* > 0.05).

Subgroup analyses were performed according to sex, age (< 70 vs. ≥ 70 years), myocardial infarction, chronic obstructive pulmonary disease, and moderate to severe chronic kidney disease to assess the robustness of the association between DIS and mortality. In the MIMIC‐IV cohort, results remained consistent across all subgroups, with DIS significantly associated with reduced 28‐day (Figure 5A) and 90‐day (Figure 5B) mortality, and no significant interactions were observed (all *p* for interaction > 0.05). Similar findings were observed in the CRHF cohort for both 28‐day (Figure 6A) and 90‐day (Figure 6B) mortality, except for a significant interaction with myocardial infarction in the 28‐day mortality analysis (*p* for interaction = 0.046).

## 4 Discussion

This multicenter study provides novel insights into the relationship between diuretic intensity and mortality outcomes in heart failure patients through the introduction and validation of the DIS system. Our findings indicate that a higher DIS was associated with lower observed 28‐ and 90‐day mortality in both Eastern and Western populations. While this association appears to challenge the conventional concern that high‐dose diuretic therapy may worsen outcomes, it must be interpreted in the context of an observational design susceptible to confounding by indication [21, 22].

The most striking finding of our study is the consistent inverse relationship between DIS and mortality across two demographically and geographically distinct cohorts. Patients in the severe DIS category (≥ 7 points) demonstrated substantially reduced mortality risk compared with those with mild DIS (1−3 points), with HRs of 0.10 and 0.27 for 28‐day mortality in the Chinese and MIMIC‐IV cohorts, respectively. This inverse association persisted at 90 days and was directionally consistent across sensitivity analyses.

Several non‐causal explanations warrant emphasis when interpreting this inverse association. First, because the DIS reflects diuretic exposure accumulated over the hospital course, patients who die very early have limited opportunity to receive escalating, combination, or intravenous regimens and are thus more likely to fall into lower‐intensity categories; this potential for survivorship and immortal‐time bias would tend to exaggerate an apparent protective effect of higher intensity. Second, treatment‐selection effects may operate strongly: clinicians often escalate diuretic therapy in patients judged to have a recoverable, congestion‐driven presentation, while withholding aggressive decongestion from those with refractory shock, advanced frailty, or comfort‐oriented goals of care, so that a higher DIS may partly capture a more favorable underlying prognosis rather than a treatment benefit. Third, despite propensity‐score matching, residual confounding by unmeasured severity markers such as left ventricular ejection fraction, NYHA class, degree of congestion, frailty, and clinician gestalt is likely; consistent with this, our E‐value analysis indicates that the association in the smaller Chinese cohort (E‐values 1.21–1.24) could be nullified by a relatively modest unmeasured confounder. Taken together, these considerations suggest that the observed association is most plausibly explained, at least in part, by selection and confounding rather than a direct protective effect of intensive diuresis.

Nevertheless, should this inverse association reflect a genuine biological effect rather than residual confounding, several mechanisms could plausibly contribute. First, higher DIS may serve as a surrogate marker for more aggressive decongestion strategies, which have been increasingly recognized as crucial for improving heart failure outcomes [4, 23, 24]. Recent evidence suggests that residual congestion at discharge is a stronger predictor of adverse outcomes than transient worsening of renal function during hospitalization [24]. These observations would be consistent with such a paradigm shift only if the association is causal; given the observational design, we cannot exclude that they instead reflect the selection and confounding mechanisms noted above [25]. Second, the DIS system inherently captures not only dose intensity but also the complexity of diuretic regimens. The use of combination therapy and intravenous administration, which contribute to higher scores, may reflect more sophisticated and individualized treatment approaches. This is particularly relevant given that our weighting algorithm assigns additional points for multidrug regimens, recognizing that combination therapy often indicates careful titration to overcome diuretic resistance rather than simple dose escalation [26].

Our findings contrast with earlier studies that reported associations between high‐dose diuretics and increased mortality [22, 27]. However, these apparent contradictions may be reconciled by considering methodological differences. Previous investigations often used crude dose categorizations or focused on single agents [28, 29], whereas our DIS system provides a more nuanced assessment of treatment intensity. Furthermore, many prior studies did not adequately account for confounding by indication—the tendency for sicker patients to receive more intensive therapy [30]. The non‐linear, dose‐dependent relationship revealed by our restricted cubic spline analysis is particularly noteworthy. While very low DIS scores were associated with increased mortality (potentially reflecting undertreatment), the inverse association plateaued at scores between 5 and 10, suggesting an optimal therapeutic window [31]. This finding resonates with the concept of personalized medicine in heart failure, where treatment intensity should be tailored to individual patient needs rather than adhering to a one‐size‐fits‐all approach [32].

If this association reflects a genuine effect rather than residual confounding, intensive diuretic therapy could operate through multiple pathways beyond simple volume removal. Effective decongestion can improve cardiac filling pressures [33], reduce wall stress [34], and enhance myocardial oxygen supply‐demand balance [35]. Additionally, relief of systemic and pulmonary congestion may reduce inflammatory activation and improve end‐organ perfusion [33, 36]. The rapid improvement in hemodynamics achieved with intravenous therapy may be particularly important in interrupting the vicious cycle of acute decompensation [37].

Strengths and Limitations. The strengths of this study include the large sample size, validation in two independent databases from distinct healthcare systems, the use of PSM with restricted cubic spline modeling, and the introduction of a standardized scoring system for diuretic intensity. Several limitations should be acknowledged. First and most importantly, the observational nature of the study precludes causal inference. Although PSM achieved acceptable covariate balance (SMDs < 0.1 for most variables), confounding by indication remains a fundamental concern: patients receiving more intensive diuretic regimens almost certainly differ from those receiving milder regimens in ways that are imperfectly captured by the measured covariates, including unmeasured markers of disease severity such as left ventricular ejection fraction, NYHA functional class, ongoing congestion, frailty, and clinician judgment regarding overall prognosis. Our E‐value analysis indicates moderate robustness in MIMIC‐IV (E‐values 1.55–1.61) but greater vulnerability in the Chinese cohort (E‐values 1.21–1.24), the latter compounded by a small number of events (*n* = 35 at 28 days, restricting Model 3 to an exploratory role). Second, we lacked data on left ventricular ejection fraction, serial natriuretic peptide measurements, and detailed hemodynamic parameters, all of which could provide mechanistic insights and reduce residual confounding. Third, the DIS does not account for dose adjustments over time, patient‐specific differences in diuretic pharmacokinetics, or pre‐admission and post‐discharge diuretic use. Fourth, important patient‐centered outcomes such as symptom burden, quality of life, and functional capacity were not available in either database. Consequently, our findings should be regarded as hypothesis‐generating associations rather than evidence of a causal benefit of intensified diuretic therapy and require validation in prospective randomized trials before any change in clinical practice.

## 5 Conclusion

In conclusion, in this propensity‐matched analysis of two independent cohorts, a higher DIS was associated with lower 28‐ and 90‐day all‐cause mortality in hospitalized heart failure patients. The DIS may serve as a useful standardized tool for characterizing diuretic intensity in observational studies and clinical audits. However, these findings should be regarded as associations and do not by themselves justify changes in clinical practice; prospective randomized trials are warranted to determine whether a DIS‐guided diuretic strategy improves clinical outcomes in acute heart failure.

## Author Contributions

**Jianwei Zhou:** conceptualization, methodology, software, formal analysis, investigation, data curation, writing – original draft, writing – review and editing, visualization.

## Ethics Statement

This study was conducted using two databases: (1) The MIMIC‐IV database, which was approved by the Institutional Review Boards of the Massachusetts Institute of Technology (MIT) and Beth Israel Deaconess Medical Center (BIDMC). The database contains only de‐identified patient information, and the requirement for individual patient consent was waived. The author completed the required Collaborative Institutional Training Initiative (CITI) program and obtained credentialed access to the database (certificate number: 65254134). (2) The China Regional Heart Failure Database from Zigong Fourth People's Hospital. This study was deemed exempt from ethical review by the institutional review board due to the retrospective nature and use of de‐identified data. This study was conducted in accordance with the Declaration of Helsinki (as revised in 2013).

## Consent

This study utilized retrospective, de‐identified data from the MIMIC‐IV database and the China Regional Heart Failure Database. Individual informed consent was waived due to the retrospective nature of the study and the use of anonymized data.

## Conflicts of Interest

The author declares no conflicts of interest.

## Data Availability

The data that support the findings of this study are available from two sources: (1) The MIMIC‐IV database (version 3.1) is publicly available and can be accessed through PhysioNet (https://physionet.org/content/mimiciv/) after completing the required training course (CITI “Data or Specimens Only Research”) and signing a data use agreement. (2) The China Regional Heart Failure Database is available from Zigong Fourth People's Hospital. Restrictions apply to the availability of these data, which were used under license for this study. Data access may be requested from the corresponding author with appropriate ethical approval and data sharing agreements. The analysis codes used in this study are available from the corresponding author upon reasonable request.
